# West Nile Virus Infection among the Homeless, Houston, Texas^1^

**DOI:** 10.3201/eid1310.070442

**Published:** 2007-10

**Authors:** Tamra E. Meyer, Lara M. Bull, Kelly Cain Holmes, Rhia F. Pascua, Amelia Travassos da Rosa, Christian R. Gutierrez, Tracie Corbin, Jennifer L. Woodward, Jeffrey P. Taylor, Robert B. Tesh, Kristy O. Murray

**Affiliations:** * University of Texas Health Science Center at Houston, Houston, Texas, USA; † University of Texas Medical Branch, Galveston, Texas, USA; ‡ Texas Department of State Health Services, Austin, Texas, USA

**Keywords:** West Nile virus, homeless, cross-sectional studies, seroepidemiologic study, enzyme-linked immunosorbent assay, immunoglobulin G, flavivirus, risk factors, dispatch

## Abstract

Among 397 homeless participants studied, the overall West Nile virus (WNV) seroprevalence was 6.8%. Risk factors for WNV infection included being homeless >1 year, spending >6 hours outside daily, regularly taking mosquito precautions, and current marijuana use. Public health interventions need to be directed toward this high-risk population.

West Nile virus (WNV) was first identified in Houston in 2002 (*1*). From 2002 through 2004, 6% of patients hospitalized with WNV infection were homeless (*2*), which raised concerns that the homeless population might be at increased risk for infection. This study was conducted to determine the seroprevalence of WNV in Houston’s homeless population after 2 transmission seasons and to determine risk factors for infection.

## The Study

A cross-sectional survey was conducted by using convenience sampling of homeless shelters, soup kitchens, homeless camps, and mobile outreach organizations. Participants gave consent and were assigned a unique study number to preserve anonymity. An interviewer-administered questionnaire collected information on demographics, social and medical histories, housing status during the 2002 and 2003 WNV transmission seasons, length of time homeless, and outdoor exposures. The study was approved by the University of Texas Health Science Center at Houston Committee for the Protection of Human Subjects (HSC-SPH-03-111).

A Mini–Mental State Examination (MMSE) was performed to evaluate the cognitive status of the participant. Participants who scored <75% on the MMSE were considered cognitively impaired, and therefore their interview responses were excluded.

As incentive, participants were provided free onsite testing and counseling for HIV, hepatitis B, hepatitis C, and WNV infections. Blood samples were collected and later tested for WNV antibodies by immunoglobulin G (IgG) ELISA and hemagglutination inhibition (HI) test. Samples were considered WNV antibody–positive if both the IgG ELISA and HI assay gave positive reactions. Data were entered into a Microsoft (Redmond, WA, USA) Access database and analyzed by using Stata 8.0 (Stata Corp., College Station, TX, USA). WNV prevalence and risk of becoming infected were calculated for each variable. Univariate odds ratios (ORs) with p<0.25 were included in a logistic regression model. A backward stepwise approach was used to eliminate variables with p>0.10 to determine a final model. Interactions between variables were assessed for significance (p<0.10), and the Hosmer-Lemeshow goodness-of-fit statistic (*3*) was used to evaluate the fit of the final model.

During the spring of 2004, 424 participants were enrolled from 13 sites; 8 were excluded due to low MMSE scores. Of the 416 participants, 397 had complete interviews, adequate blood samples, and were included in the analysis. This sampling represents ≈4% of Houston’s estimated 10,000 homeless population (*4*).

Of the 397 participants, 27 were WNV positive (seroprevalence 6.8%; 95% confidence interval [CI] 4.5–9.7). Men represented 72% of the participants, with 8.4% found to be positive for WNV, compared with 2.7% of women (OR 3.3; 95% CI 0.96–11.0) (Table 1). The study population was 59% black, 30% white, and 11% “other” or not stated; 13% were of Hispanic ethnicity. Mean age was 42 years (range 18–69 years).

**Table 1:** Participant demographics and WNV prevalence from the 2004 Houston Homeless Seroprevalence Study*

Demographic characteristics	All participants, n = 397 (%)		WNV prevalence			Risk for WNV infection, OR (95% CI)
			No. (%)	95% CI		
Sex						
Female	110 (28)		3 (2.7)	0.6–7.8		Reference
Male	287 (72)		24 (8.4)	5.4–12.2		3.3 (0.96–11.0)
Age, y						
18–34	97 (24)		2 (2.1)	0.3–7.3		Reference
35–49	204 (51)		17 (8.3)	4.9–13.0		4.3 (0.98–19.1)
>50	95 (24)		8 (8.4)	3.7–15.9		4.4 (0.9–21.1)
Unknown	1 (0.3)		0	–		–
Race						
White	120 (30)		8 (6.7)	2.9–12.7		Reference
Black	233 (59)		18 (7.7)	4.6–11.9		1.2 (0.5–2.8)
Other/unknown	44 (11)		1 (2.3)	0.06–12.0		0.3 (0.04–2.7)
Ethnicity						
Hispanic	52 (13)		2 (3.9)	0.5–13.2		Reference
Non-Hispanic	343 (86)		24 (7.0)	4.5–10.2		1.9 (0.4–8.2)
No response	2 (0.5)		1 (50.0)	1.3–98.7		–

*WNV, West Nile virus; OR, odds ratio; CI, confidence interval.

For both 2002 and 2003 transmission seasons, 278 (70%) participants reported having stable housing, and WNV seroprevalence was 4.7% (95% CI 2.5–7.9) (Table 2). For those who had unstable housing in both 2002 and 2003 (n = 45; 11%), we found a significantly higher WNV seroprevalence of 13.3% (OR 3.1, 95% CI 1.1–8.7). For those who reported being homeless >1 year (n = 73; 18%), seroprevalence for WNV was 16.4% (95% CI 8.8–27.0), with a significantly increased risk for WNV infection when compared with those who did not consider themselves homeless or were homeless <1 month (OR 3.2; 95% CI 1.3–7.7, p = 0.01). When asked about the average length of time spent outdoors during the summer and fall, 38% reported <6 hours per day (seroprevalence 2.0%), 38% reported >6–12 hours (seroprevalence 8.0%), and 24% reported >12 hours (seroprevalence 12.5%). There was a positive trend (p value for trend 0.002) between number of hours spent outside and increased risk for WNV infection. Current marijuana use was also associated with WNV infection (OR 2.5; 95% CI 1.0–6.0).

**Table 2:** Self-reported social histories and prevalence of WNV infection from the 2004 Houston Homeless Seroprevalence Study*

Participant characteristics	All participants, n = 397 (%)		WNV prevalence			Risk for WNV infection, OR (95% CI)
			No. (%)	95% CI		
Housing status†						
Stable housing, 2002 and 2003	278 (70)		13 (4.7)	2.5–7.9		Reference
Unstable housing, 2002 or 2003	69 (17)		7 (10.1)	4.2–19.8		2.3 (0.9–6.0)
Unstable housing, 2002 and 2003	45 (11)		6 (13.3)	5.1–26.8		3.1 (1.1–8.7)‡
Unknown	5 (1)		1 (20.0)	0.5–71.6		–
Homelessness status						
Does not consider himself or herself homeless	111 (28)		8 (7.2)	3.2–13.7		Reference
Lives mostly on the streets	50 (13)		6 (12.0)	4.5–24.3		1.8 (0.6–5.4)
Lives in temporary shelter	125 (31)		10 (8.0)	3.9–14.2		1.1 (0.4–2.9)
Lives temporarily with friends/family	64 (16)		1 (1.6)	0.04–8.4		0.2 (0.02–1.7)
Other	47 (12)		2 (4.3)	0.5–14.5		0.6 (0.1–2.8)
Length of time homeless						
Does not consider himself or herself homeless or homeless <1 mo	171 (43)		10 (5.9)	2.8–10.5		Reference
1 mo–1 y	153 (39)		5 (3.3)	1.1–7.5		0.5 (0.2–1.6)
>1 y	73 (18)		12 (16.4)	8.8–27.0		3.2 (1.3–7.7)‡
Time spent outdoors on average each day during summer and fall§						
<6 h	150 (38)		3 (2.0)	0.4–5.7		Reference
6–12 h	150 (38)		12 (8.0)	4.2–13.6		4.3 (1.2–15.4)¶
>12 h	96 (24)		12 (12.5)	6.6–20.8		7.0 (1.9–25.5)‡
Unknown	1 (0.3)		0 (0)	–		–
Substance use#						
Current tobacco use	273 (69)		20 (7.3)	4.5–11.1		1.3 (0.5–3.2)
>15 drinks containing alcohol/wk	70 (18)		3 (4.3)	0.9–12.0		0.6 (0.2–1.9)
Ever used street drugs	281 (71)		19 (6.8)	4.1–10.4		1.0 (0.4–2.3)
Ever used needles to inject street drugs	89 (22)		6 (6.7)	2.5–14.1		1.0 (0.4–2.5)
Current drug use (within past 6 mo)	108 (27)		11 (10.2)	5.2–17.5		1.9 (0.9–4.3)
Street drugs used in past 6 mo**						
CNS stimulants (crack/cocaine/ amphetamines)	84 (21)		5 (6.0)	2.0–13.3		0.8 (0.3–2.3)
Heroin/opiates	4 (1)		0 (0)	–		–
Marijuana	61 (15)		8 (13.1)	5.8–24.2		2.5 (1.0–6.0)¶

*WNV, West Nile virus; OR, odds ratio; CI, confidence interval; CNS, central nervous system. †p value for trend = 0.02. ‡Significant at α = 0.01. §p value for trend = 0.002. ¶Significant at α = 0.05. #Alcohol and current drug use will most likely be underestimated because shelters did not allow use of these substances. **Not mutually exclusive, hence univariate analysis compared specific drug use with no use of that drug.

Univariate analysis identified the following variables as significantly (α<0.05) associated with risk for WNV infection: unstable housing in 2002 and 2003, being homeless >1 year, spending >6 hours outside per day during the summer and fall, and current marijuana use. The final logistic regression model identified the following independent risk factors (p<0.10) for WNV infection: being homeless >1 year (OR 3.8, p = 0.002), spending >6 hours outdoors (OR 4.3, p = 0.02), normally taking mosquito precautions (OR 2.8, p = 0.04), and current marijuana use (OR 2.4, p = 0.07). The Hosmer-Lemeshow goodness-of-fit-test statistic was 12.4 (p>0.19), which suggests that the model is a good fit. When interaction terms were entered into the model, the interaction between marijuana smoking and spending >6 hours outdoors was significant (likelihood ratio p = 0.04) and increased the strength of the association with WNV infection.

## Conclusions

We believe this is the first study to determine the prevalence of WNV in homeless adults and to determine risk factors for becoming infected among this high-risk urban population. Findings from this study will help public health authorities determine appropriate intervention and prevention strategies.

We found a seroprevalence of 6.8% in our sample of homeless persons, with a seroprevalence of 16.4% in persons reporting being homeless >1 year. Other studies have assessed the prevalence of WNV in general populations in the United States (*5*–*10*), with estimates of 0%–14%.

To our knowledge, this is the first report of WNV seroprevalence in a population with high-risk outdoor exposures. Only 3 studies have evaluated risk factors for infection in the United States and found that increased time outdoors (*5*,*8*), inconsistent use of mosquito repellant (*5*), and age (*9*) were predictors for infection. In Houston’s homeless population, spending >6 hours outside per day during the summer and fall and being homeless >1 year independently predicted risk for infection. Although being homeless >1 year was highly associated with increased time spent outdoors, this variable also independently predicted infection. This finding is important in a public health context because it highlights a strong potential for further cases of WNV infection in this population.

We found that regularly using mosquito precautions was associated with an increased risk for infection, which differs from the findings in New York (*5*). This finding was surprising since, in theory, use of mosquito precautions should reduce the risk for WNV infection. However, when asked about the types of mosquito precautions used, many participants reported methods that may be ineffective such as using candles or fire as a deterrent or swatting at mosquitoes. Education regarding appropriate preventive methods would be valuable in this population.

In addition, we found that marijuana use predicted WNV infection, which is difficult to explain. To our knowledge, this is the first report of marijuana use being a risk factor for WNV infection. Several explanations are possible, however: 1) this finding was due to chance, 2) persons using marijuana may spend more time outdoors between dusk and dawn when the *Culex* mosquito is most active, 3) the mosquito vector could be attracted to marijuana smoke, or 4) marijuana use could affect cognition, thereby preventing the user from interrupting a mosquito taking a blood meal. The relationship between marijuana use and WNV infection deserves further investigation.

For comparison, data on WNV prevalence in a nonhomeless population during the same time period and location would be useful. After the 2003 transmission season, a study at the University of Texas Health Science Center at Houston found a seroprevalence of 4.7% among 274 students, faculty, and staff (K. Murray, unpub. data).

This study provides important information on the magnitude and risk factors for WNV infection among homeless persons. Combining education with distribution of effective mosquito prevention aids such as mosquito repellent may help reduce the risk for WNV infection and other mosquitoborne diseases in this high-risk population.
